# Integrative miRNA–mRNA Network and Molecular Dynamics-Based Identification of Therapeutic Candidates for Paroxysmal Nocturnal Hemoglobinuria

**DOI:** 10.3390/ph19010143

**Published:** 2026-01-14

**Authors:** Peng Zhao, Yujie Tang, Xin Sun, Yibo Xi, Haojun Zhang, Jia Xue, Wenqian Zhou, Hongyi Li, Xuechun Lu

**Affiliations:** 1 School of Management, Shanxi Medical University, Taiyuan 030000, China; peng_zhao_919@163.com (P.Z.); tangyujie3816@163.com (Y.T.);; 2 Medical School of Chinese PLA, Beijing 100000, China; 3 Department of Hematology, Second Medical Center, Chinese PLA General Hospital, Beijing 100000, China; 4 National Clinical Research Center for Geriatric Diseases, Beijing 100000, China

**Keywords:** paroxysmal nocturnal hemoglobinuria, miRNA–mRNA regulatory network, drug repositioning, scRNA seq, molecular docking, molecular dynamics simulation

## Abstract

**Background:** Paroxysmal nocturnal hemoglobinuria (PNH) is a clonal hematopoietic stem cell disease characterized primarily by intravascular hemolysis, thrombosis, and bone marrow failure. Complement inhibitors are commonly used in clinical treatment and show limited efficacy, highlighting the urgent need to identify new therapeutic targets and explore alternative treatment strategies to provide theoretical guidance for clinical practice. **Methods:** We established a PNH cell model and constructed an miRNA–mRNA regulatory network to identify key miRNAs and core target genes. Single-cell sequencing data were analyzed to further clarify the critical genes. Finally, integrated drug database analysis identified potential therapeutic agents for PNH, which were validated by molecular docking and molecular dynamics simulations. **Results:** Using CRISPR/RNP technology, we successfully constructed a *PIGA*-knockout (*PIGA*-KO) THP-1 cell model. Differential expression analysis identified 1979 differentially expressed mRNAs (DEmRNAs) and 97 differentially expressed miRNAs (DEmiRNAs). The multiMiR package in R was used to predict the target genes of DEmiRNAs, from which those experimentally validated through dual-luciferase reporter assays were selected. After integration with the DEmRNAs, an miRNA–mRNA regulatory network was constructed, comprising 26 miRNAs and 38 mRNAs. Subsequent miRNA pathway enrichment analysis identified hsa-miR-23a-3p as a key miRNA, with *CXCL12*, *CXCL8*, *HES1*, and *TRAF5* serving as core target genes. The integration of single-cell sequencing datasets (PRJNA1061334 and GSE157344) was performed, followed by cell communication and enrichment analysis. This approach, combined with clinical relevance, identified the neutrophil cluster as the key cluster. Intersection analysis of neutrophil cluster differential analysis results with key modules from hdWGCNA further clarified the critical genes. Drug prediction using EpiMed, CMap, and DGIdb identified Leflunomide, Dipyridamole, and Pentoxifylline as potential therapeutic agents. Molecular docking and molecular dynamics simulations showed stable binding of these potential drugs to the critical molecules, indicating a viable molecular interaction foundation. **Conclusions:** Leflunomide, Dipyridamole, and Pentoxifylline may serve as promising therapeutic agents for PNH, and the hsa-miR-23a-3p/*CXCL8* regulatory axis could play a pivotal role in the pathogenesis and progression of PNH.

## 1. Introduction

Paroxysmal nocturnal hemoglobinuria (PNH) is a rare acquired clonal hematopoietic stem cell disorder. The core pathological mechanism involves somatic mutations in *PIGA* in hematopoietic stem cells, leading to impaired synthesis of glycosylphosphatidylinositol-anchored proteins (GPI-APs). Red blood cells lacking GPI-APs are highly susceptible to complement-mediated attack, resulting in intravascular hemolysis, paroxysmal hemoglobinuria, bone marrow failure, and venous thrombosis. In some cases, patients may also develop hematologic malignancies [1].

With the progress in research and applications in complement C5 inhibitors such as Eculizumab [2], Pozelimab [3], and Crovalimab [4], complement C3 inhibitors like Pegcetacoplan [5,6], complement factor D inhibitors like Danicopan [7], and complement factor B inhibitors such as Iptacopan [8], the survival rate and quality of life of patients have significantly improved. However, current drug therapies still have a number of limitations, including high drug costs, variable efficacy among different patients, infection risks associated with long-term use, compliance issues, and the development of drug resistance in some patients [9,10]. Therefore, the development of safer, more effective, and cost-efficient therapeutic drugs to continuously improve benefits for PNH patients has become a critical issue to addressed.

Drug repositioning (DR) refers to a strategy for identifying new uses for existing drugs that have been approved for other medical indications or are still in the experimental phase [11]. This approach not only reduces the time and cost associated with new drug development but also significantly increases the success rate of repurposing for new indications, owing to the extensive existing data on drug safety, pharmacokinetics, and other factors [12,13]. Moreover, with the rapid development of computational biology and artificial intelligence technologies, methods such as molecular docking (MD) and molecular dynamics simulation (MDS) are now widely applied in DR research [14,15]. MD enables precise prediction of the binding modes and affinities between drugs and target proteins, facilitating high-throughput drug screening [16]. MDS further reveals the stability and mechanism of action of drug–protein complexes, providing atomic-level insights into their interactions and offering a theoretical foundation for subsequent experimental validation [17]. These technological approaches not only accelerate the drug screening process but also provide powerful tools for developing an in-depth understanding of drug mechanisms and optimizing the structure of candidate molecules. Therefore, the strategy of combining DR with MD and MDS offers a new pathway for the development of potential therapeutic drugs for rare diseases such as PNH, which have a well-defined pathogenesis and urgently need to be treated but which have a relatively small patient population. However, the effectiveness of DR strategies critically depends on the identification of biologically relevant and disease-associated targets. In this context, microRNAs (miRNAs) have emerged as important regulatory nodes linking dysregulated gene networks to druggable protein targets. By modulating the expression of multiple genes simultaneously, miRNAs can influence inflammatory signaling, immune responses, and thrombotic pathways, thereby providing a mechanistic bridge between systems-level disease regulation and structure-based drug discovery.

miRNAs are a class of endogenous non-coding small RNAs that regulate gene expression by binding to the 3′-UTR of target mRNAs, leading to mRNA degradation or translation repression. As key regulators of gene expression, miRNAs have been attracting increasing attention for their role in hematological diseases such as PNH [18]. They also play a significant role in various biological processes, including cell proliferation, differentiation, and apoptosis [19]. Studies have shown that specific miRNAs may be involved in the pathogenesis of PNH by modulating complement systems, hematopoietic stem cell functions, and the immune microenvironment [20]. Additionally, miRNAs have emerged as novel therapeutic targets, with evidence suggesting that their expression levels can be modulated using drugs, thereby influencing downstream signaling pathways and providing new insights to inform the treatment of rare diseases such as PNH [21,22]. In drug repositioning research, drug screening and mechanistic exploration based on miRNA regulatory networks represent an emerging area of interest. By integrating miRNA-related target information, it is possible to further enrich the drug target spectrum and enhance the efficiency of candidate drug screening.

**Figure 1:**
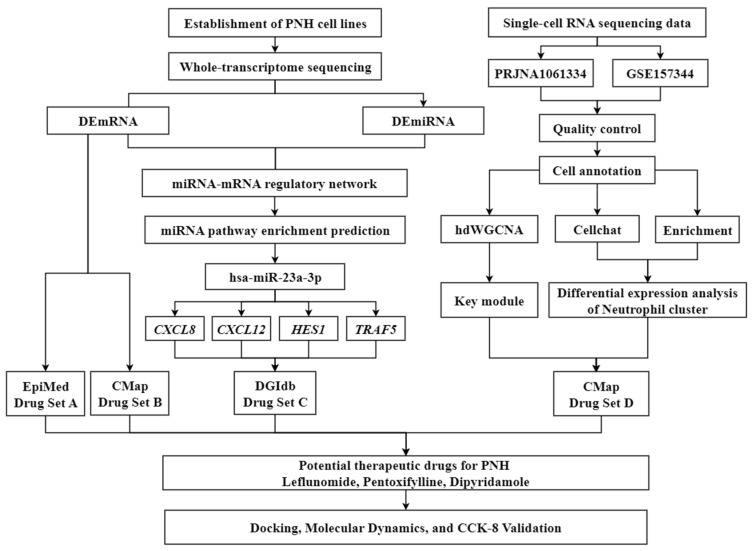
Overall workflow of the present study.

In light of this, the present study focused on DR for PNH, aiming to identify potential therapeutic agents for precision treatment in PNH and elucidate the theoretical basis behind them. Firstly, a systematic review of PNH-related molecular targets was conducted, and MD was used to screen FDA-approved drugs that have good binding affinity with PNH targets. Next, MDS was employed to analyze the structural stability and interaction mechanisms of the drug–target complexes. Finally, through the integration of pharmacological information, the feasibility of the candidate drugs for clinical treatment of PNH was explored (Figure 1). This study not only provides potential drugs and personalized treatment options for PNH patients but also offers new theoretical insights into the application of DR in the field of rare hematological diseases.

## 2. Results

### 2.1. Establishment and Validation of PNH Cell Lines

After electroporating THP-1 cells with the CRISPR/Cas9 RNP system, a *PIGA*-KO cell line was successfully obtained. RT-qPCR validation showed that the expression level of *PIGA* in the *PIGA*-KO cell line was significantly lower than in the WT cell line (Supplementary Figure S1, Supplementary Table S1). The obtained monoclonal knockout clones maintained a stable *PIGA*-deficient phenotype during long-term culture.

### 2.2. Differential Analysis and Enrichment Analysis

**Figure 2:**
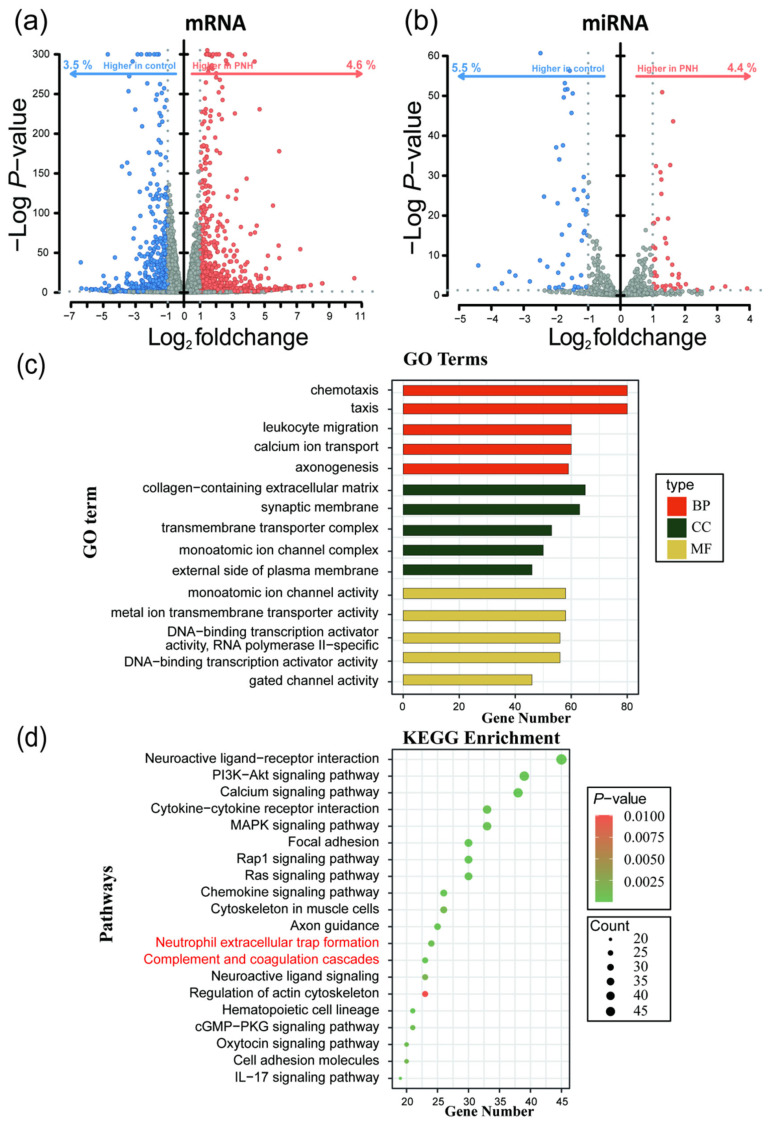
Differential expression analysis and GO/KEGG enrichment analysis of the whole-transcriptome sequencing data. (**a**) Volcano plot of differential expression analysis of mRNA. (**b**) Volcano plot of differential expression analysis of miRNA. (**c**) GO functional enrichment analysis of mRNA. (**d**) KEGG functional enrichment analysis of mRNA.

Differential expression analysis of mRNA sequencing data was performed using the DESeq2 package in R 4.4.1, resulting in 1979 DEmRNAs, of which 1117 were upregulated and 862 were downregulated. Differential analysis of miRNA sequencing data identified 97 DEmiRNAs, with 43 upregulated and 54 downregulated (Figure 2a,b, Supplementary Table S2).

Gene Ontology (GO) and Kyoto Encyclopedia of Genes and Genomes (KEGG) enrichment analyses were conducted on the differentially expressed genes (DEGs). GO enrichment analysis revealed that the DEGs were enriched in 1116 pathways, with 161 related to molecular functions (MFs), 105 related to cellular components (CCs), and 850 related to biological processes (BPs). Enriched pathways included “calcium ion transport”, “regulation of monoatomic ion transport”, and “metal ion transmembrane transporter activity”, which are mainly associated with immune response, ion transport and signaling, and gene expression regulation. KEGG enrichment analysis showed that the DEGs were enriched in 58 pathways (Supplementary Table S3), including “Hematopoietic cell lineage”, “Neutrophil extracellular trap formation”, and “Complement and coagulation cascades”, which are primarily involved in signaling transduction, cell communication, hematopoiesis and coagulation, and complement system functions (Figure 2c,d).

### 2.3. Construction of miRNA-mRNA Regulatory Axis and Identification of Key Genes

**Figure 3:**
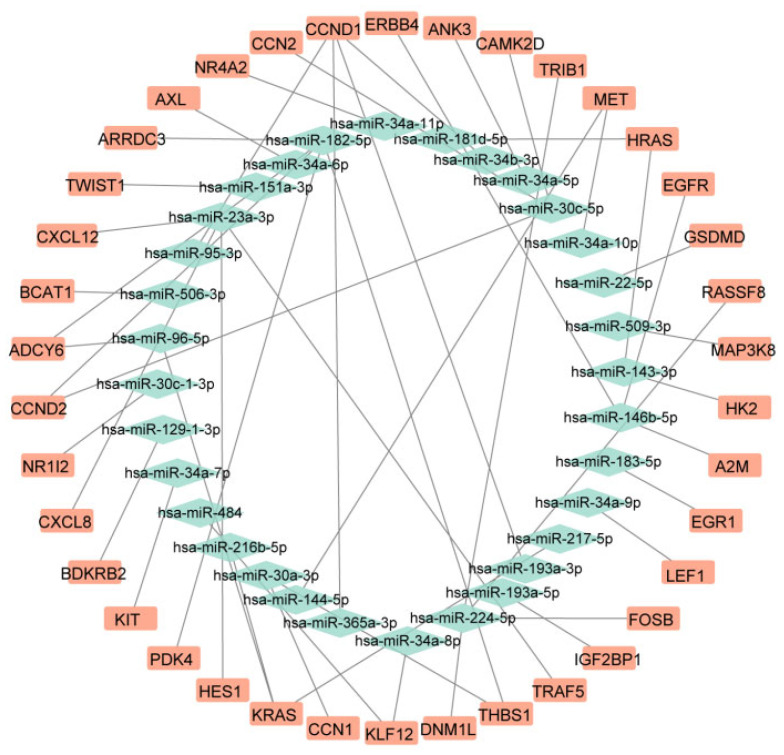
miRNA-mRNA Regulatory Network.

Target gene prediction was performed using the multiMiR package in R (version 4.1.1). Only miRNA–mRNA interactions experimentally validated via dual-luciferase reporter assays were retained, resulting in 66 miRNAs corresponding to 638 potential target genes (Supplementary Table S4). To identify biologically meaningful regulatory relationships, target genes of upregulated miRNAs were intersected with downregulated mRNAs, while target genes of downregulated miRNAs were intersected with upregulated mRNAs. Based on this strategy, an miRNA–mRNA regulatory network consisting of 26 miRNAs and 38 mRNAs was constructed (Figure 3, Table 1).

Subsequent miRNA pathway enrichment analysis revealed that only hsa-miR-23a-3p was significantly enriched in PNH-related pathways, including KEGG_Complement and Coagulation Cascades, KEGG_Hematopoietic Cell Lineage, and KEGG_Glycosylphosphatidylinositol (GPI) Anchor Biosynthesis (Supplementary Table S5). These findings support the identification of hsa-miR-23a-3p as a core candidate miRNA in PNH, with *CXCL12*, *CXCL8*, *HES1*, and *TRAF5* emerging as key target genes within the PNH-associated regulatory network. Specifically, the Log_2_FC for these genes were as follows: miR-23a-3p, 1.1; *CXCL12*, −1.28; *CXCL8*, −1.2; *HES1*, −1.8; and *TRAF5*, −1.49. These differential expression values further highlight the potential impact of these molecules in the pathogenesis of PNH.

To further explore the regulatory relationships between hsa-miR-23a-3p and its target genes, Pearson correlation analysis was performed using matched miRNA-seq and mRNA-seq data from the same samples (Supplementary Table S6). The results revealed context-dependent miRNA–mRNA associations. For example, hsa-miR-23a-3p and *CXCL8* showed a positive correlation in WT samples, whereas an inverse correlation trend was observed in *PIGA*-KO samples. When all matched samples (n = 6) were analyzed collectively, a significant overall correlation was observed.

These findings indicate that miRNA–mRNA relationships may vary depending on both within-group variability and between-group effects. While differential expression analysis reflects average expression differences between experimental groups, correlation analysis captures sample-level co-variation. Therefore, opposite differential expression patterns between groups do not necessarily imply a negative correlation within each group. Given the indirect and context-dependent nature of miRNA-mediated regulation, the observed correlations should be interpreted as supportive but not definitive evidence of functional interaction.

### 2.4. Single-Cell Sequencing Data Analysis

**Figure 4:**
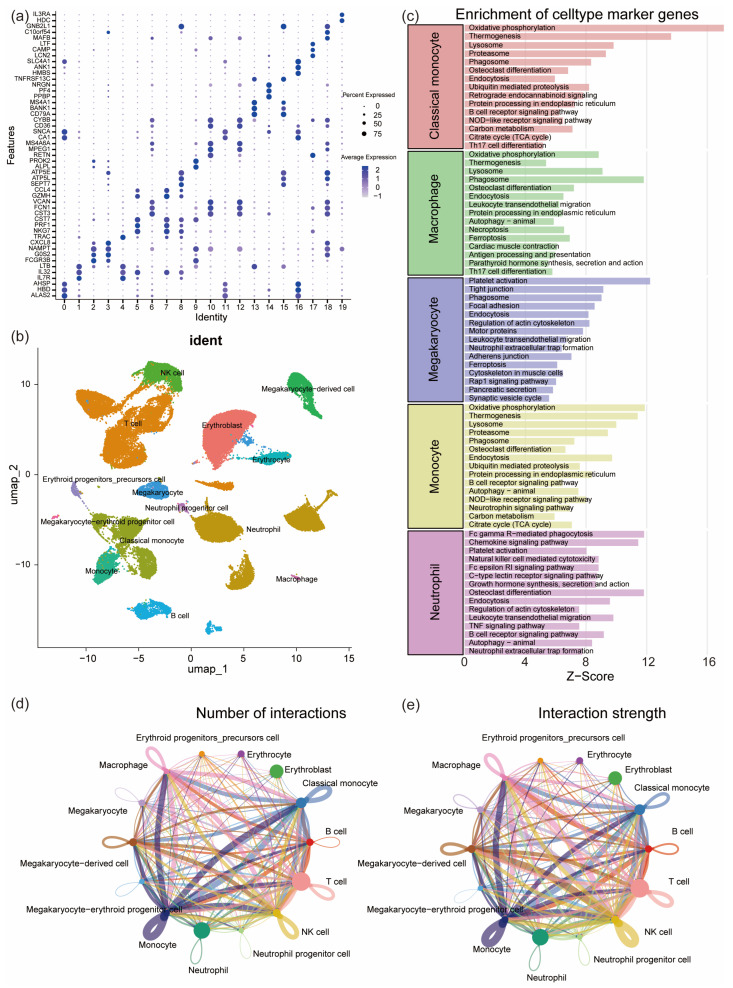
Single-cell data analysis results. (**a**) A dot plot showing the expression of typical marker genes across different cell subpopulations. The size of the dots represents the expression proportion, and the color represents the average expression level. (**b**) UMAP dimensionality reduction clustering plot displaying the distribution of major cell types, including monocytes, macrophages, neutrophils, NK cells, B cells, T cells, erythroid lineage, and megakaryocyte lineage cells, after annotation using multiple databases. (**c**) Functional pathway enrichment analysis of marker genes for different cell types, with a bar plot showing the characteristic pathways of each cell type and their enrichment Z-scores. (**d**) Cell interaction count analysis, with a chord diagram showing the number of interactions between different cell types, where the width of the connecting lines represents the number of interactions. (**e**) Cell interaction strength analysis, with a chord diagram showing the interaction strength between different cell types, where the width of the connecting lines represents the strength of the interactions.

The disease group data were obtained from the dataset PRJNA1061334 (https://www.ncbi.nlm.nih.gov/bioproject/?term=PRJNA1061334, accessed on 5 August 2025) and the control group data from GSE157344 (https://www.ncbi.nlm.nih.gov/geo/query/acc.cgi?acc=GSE157344, accessed on 5 August 2025). Quantification of the disease group was performed using Cell Ranger 9.0.1 software with default parameters. The disease and control group data were integrated and analyzed using the Seurat (version 5.2.0) and Harmony (version 1.2.4) packages in R 4.1.1. After annotating the cells using multiple databases, 14 cell types were identified, including monocytes, erythrocytes, neutrophils, T cells, B cells, and megakaryocytes (Figure 4a,b). Enrichment analysis of the monocyte cluster, macrophage cluster, megakaryocyte cluster, and neutrophil cluster showed that neutrophils were enriched in pathways such as “Platelet activation” and “Neutrophil extracellular trap formation” (Figure 4c). Cell communication analysis revealed that neutrophils had larger nodes and multiple thick lines connecting them to various immune cells, indicating their high proportion in the single-cell dataset and their central and highly active role in the cell communication network (Figure 4d,e). Therefore, a differential expression analysis (*p* < 0.05) was performed on the neutrophil cluster, resulting in 7700 differentially expressed genes, with 5670 upregulated and 2030 downregulated (Supplementary Table S7, Supplementary Figure S2). Notably, the expression of the *PIGA* gene was significantly downregulated in neutrophils in the disease group, further supporting its potential role in the pathogenesis of PNH (Supplementary Figure S3).

**Figure 5:**
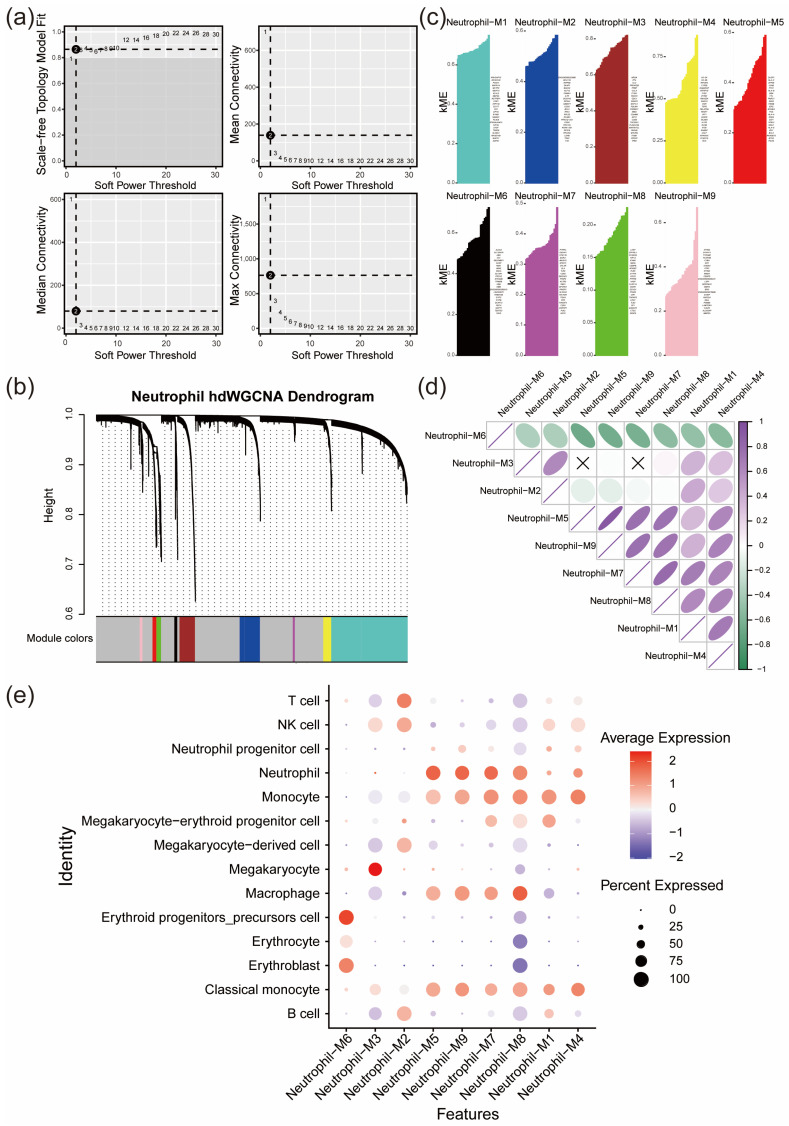
hdWGCNA analysis results. (**a**) Plot showing the soft threshold power determination used in hdWGCNA, with 12 selected as the optimal threshold. (**b**) Dendrogram generated by WGCNA displaying the hierarchical clustering of genes into different modules based on co-expression patterns. (**c**) Bar plot showing the module characteristic gene (kME) values for genes within different color-coded modules (black, blue, brown, green, magenta, pink, red, turquoise, yellow). (**d**) Correlation analysis illustrating the relationships between different gene modules, with positive and negative correlations represented in blue and green, respectively. (**e**) Dot plot showing the correlation between different cell clusters and key modules.

hdWGCNA analysis was performed on the integrated single-cell data with a soft threshold of 12, selected based on the scale-free topology criterion. This value was chosen as it was the lowest power at which the scale-free topology model fit (signed R^2^) exceeded 0.9, while still maintaining adequate network connectivity, in accordance with the standard WGCNA/hdWGCNA framework. The results revealed that the single-cell objects could be divided into nine modules: black, blue, brown, green, magenta, pink, red, turquoise, and yellow. Modules 5, 7, and 9, corresponding to the red, magenta, and pink modules, respectively (Figure 5), exhibited strong correlations with the neutrophil cluster and were therefore selected as key modules. Genes from the key modules were extracted and intersected with the DEGs in the neutrophil cluster, resulting in 154 key neutrophil genes, with 79 upregulated and 75 downregulated, which were used for subsequent potential drug prediction (Supplementary Table S8).

### 2.5. Potential Drug Prediction for PNH

**Figure 6:**
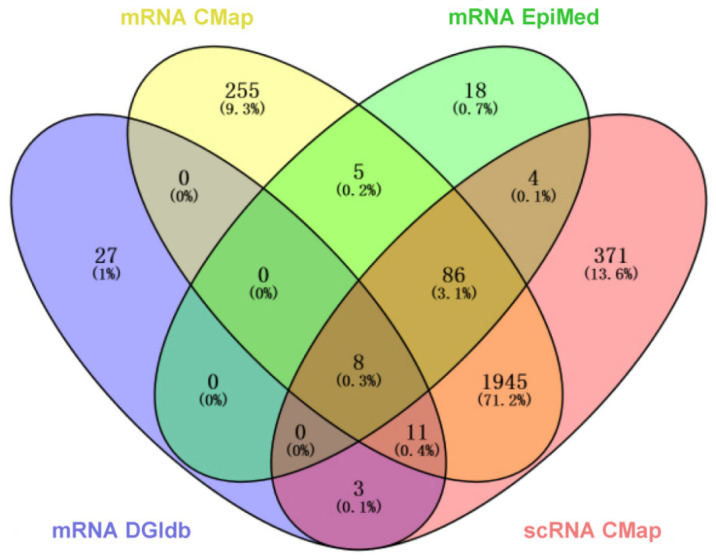
Venn diagram of drug sets A (mRNA EpiMed), B (mRNA CMap), C (mRNA DGIdb), and D (scRNA CMap).

DEmRNAs were input into EpiMed for drug prediction (*p* < 0.05, Correlation < −0.1), yielding drug set A. DEmRNAs were also input into the CMap database for drug prediction (norm_cs < 0), resulting in drug set B. The four core genes (*CXCL12*, *CXCL8*, *HES1*, *TRAF5*) were input into DGIdb for drug prediction (Drug Status: Approved), producing drug set C. Key neutrophil cluster genes were input into the CMap database for drug prediction (norm_cs < 0), resulting in drug set D. The intersection of drug sets A, B, C, and D revealed eight candidate drugs: Prednisone (PDN), Tretinoin (ATRA), Dipyridamole (DIP), Danazol (DAN), Pentoxifylline (PTX), Leflunomide (LEF), Colchicine (COL), and Paclitaxel (Figure 6, Table 2, Supplementary Table S9). Prednisone, as a corticosteroid, has already been included in PNH treatment guidelines; Tretinoin has been confirmed to have potential therapeutic effects for PNH; Danazol, a synthetic androgen, has significant hepatic burden; and Colchicine and Paclitaxel, as chemotherapeutic agents, were not considered in this study. Leflunomide (LEF), as an immunomodulatory drug, can suppress inflammation, reduce complement-mediated hemolysis, and prevent thrombosis; Dipyridamole (DIP), an anti-platelet aggregation drug, can inhibit platelet activation and aggregation and expand blood vessels and has a low risk of bleeding and minimal side effects; Pentoxifylline (PTX), an agent that improves microcirculation, can reduce blood viscosity, enhance red blood cell deformability, and inhibit platelet aggregation. Therefore, through this study, we identified LEF, DIP, and PTX as potential therapeutic drugs for PNH.

### 2.6. Molecular Docking Results

**Figure 7:**
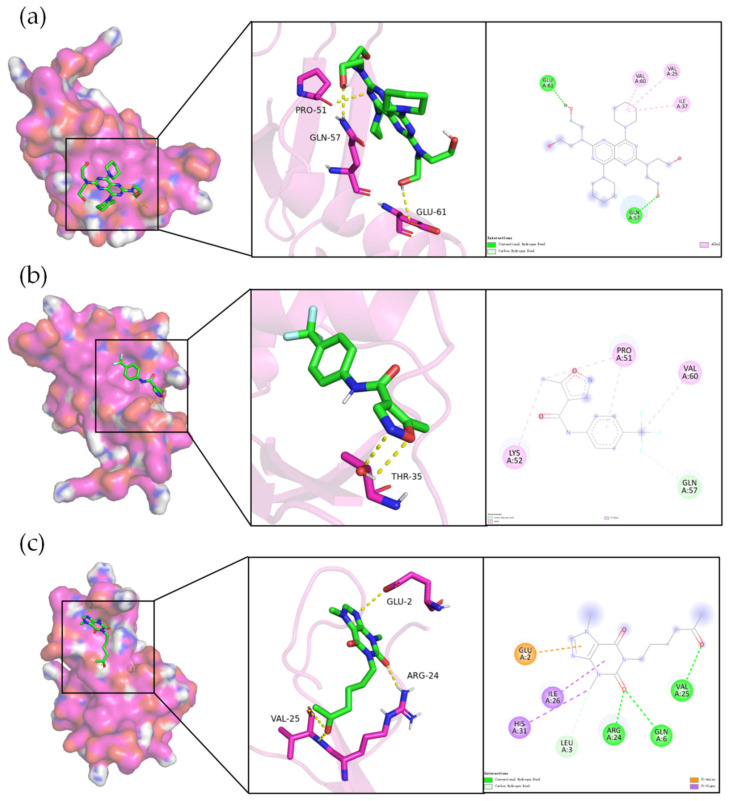
MD results of DIP, LEF, and PTX binding with CXCL8. (**a**) Binding mode of DIP with CXCL8. (**b**) Binding mode of LEF with CXCL8. (**c**) Binding mode of PTX with CXCL8.Colors represent different interaction types, including conventional hydrogen bonds (green), carbon hydrogen bonds (light green), alkyl interactions (pink), π–sigma interactions (purple), and π–anion interactions (orange).

The molecular dynamics (MD) simulation results for DIP, LEF, and PTX in complex with CXCL8 (PDB ID:6N2U), visualized using PyMOL 3.1 (Figure 7), reveal that all three drugs exhibit promising interactions with the CXCL8 protein. The binding energies for these complexes are approximately −4.8 kcal/mol for DIP, −6.0 kcal/mol for LEF, and −4.9 kcal/mol for PTX, suggesting relatively modest but consistent binding across all three drugs. These findings indicate the potential for interaction, which aligns with the molecular docking predictions. While the binding affinities are moderate, they suggest that DIP, LEF, and PTX could bind to CXCL8, albeit weakly, and that further optimization of their binding affinity may be possible.

Detailed analysis of the interactions shows that each drug engages with key residues at the CXCL8 binding site. DIP forms hydrogen bonds with PRO-51, GLN-57, and GLU-61, and π-π interactions between aromatic side chains contribute to stabilizing the complex. LEF interacts primarily through hydrogen bonds with THR-35 and is further stabilized by salt-bridge interactions, indicating a combination of polar and non-polar interactions. PTX, on the other hand, interacts with VAL-25, GLU-2, and ARG-24, forming hydrogen bonds and hydrophobic interactions, particularly with VAL-25, suggesting that PTX fits well within the hydrophobic regions of CXCL8.

While these interactions show potential for binding, the relatively weak binding energies observed suggest that these complexes may not be stable enough for long-term, high-affinity binding. The absence of strong hydrogen bonds or stable π-π interactions, typically seen in high-affinity ligands, indicates that the interactions are not yet optimal. Nevertheless, the results highlight that these drugs do have the potential to interact with CXCL8, with room for further enhancement in binding strength.

### 2.7. Molecular Dynamics Simulation Results

**Figure 8:**
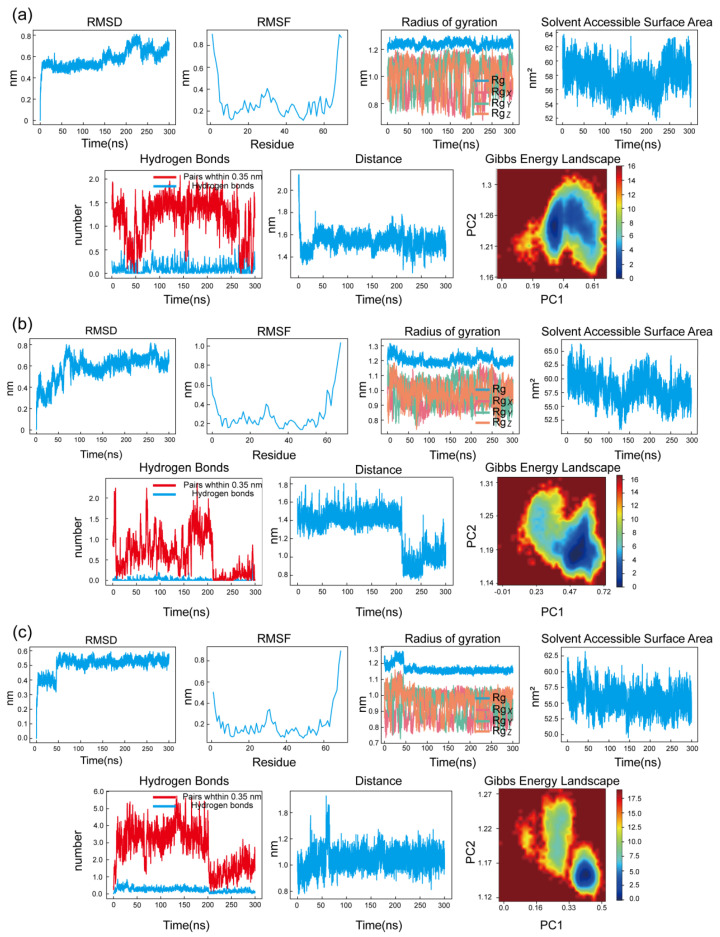
MDS results of the DIP, LEF, and PTX-CXCL8 complexes. (**a**) RMSD, RMSF, Rg, SASA, Hydrogen Bonds, Interatomic Distance and FEL of the DIP-CXCL8 complex. (**b**) RMSD, RMSF, Rg, SASA, Hydrogen Bonds, Interatomic Distance and FEL of the LEF-CXCL8 complex. (**c**) RMSD, RMSF, Rg, SASA, Hydrogen Bonds, Interatomic Distance and FEL of the PTX-CXCL8 complex. Each group displays the system stability, flexibility changes, conformational compactness, and energy distribution characteristics.

Molecular dynamics (MD) simulations were performed following semi-flexible docking, which does not fully account for protein conformational flexibility. To further evaluate the dynamic stability and binding behavior of the complexes, 300 ns MD simulations were conducted for the LEF–CXCL8, DIP–CXCL8, and PTX–CXCL8 systems (Figure 8).

The RMSD trajectories of all three complexes exhibited an initial increase during the early stages of the simulations, corresponding to system relaxation, followed by the emergence of stable plateaus, indicating that equilibrium was achieved. Specifically, the DIP–CXCL8 and PTX–CXCL8 complexes reached equilibrium after approximately 50 ns, whereas the LEF–CXCL8 complex required a longer equilibration period and stabilized after approximately 100 ns.

Following equilibration, RMSD fluctuations remained within a narrow range for each system, suggesting the absence of major conformational rearrangements during the production phase. These observations indicate that all three protein–ligand complexes maintained stable global conformations throughout the equilibrated portion of the 300 ns simulations.

Consistent with the RMSD results, RMSF analysis revealed limited residue-level flexibility, mainly localized to loop and terminal regions, while the core regions of the protein remained structurally stable. Such localized fluctuations are expected and reflect the intrinsic flexibility necessary for protein function and ligand accommodation.

The radius of gyration (Rg) profiles remained relatively constant after equilibration, indicating that the overall compactness of the protein structures was preserved and that no significant unfolding or collapse occurred during the simulations. Similarly, solvent-accessible surface area (SASA) values exhibited only minor fluctuations, further supporting the stability of the protein–solvent interface.

Hydrogen bond analysis demonstrated persistent and stable hydrogen-bonding interactions between CXCL8 and the ligands throughout the equilibrated phase of the simulations. The number of hydrogen bonds remained largely unchanged, indicating sustained intermolecular interactions that contribute to binding stability.

Interatomic distance analysis further confirmed that key ligand–protein contacts were preserved over time, supporting a consistent binding mode within the active site after equilibration. Moreover, Gibbs free energy landscape (FEL) analysis revealed a well-defined low-energy basin for each complex, with a dominant minimum energy state, suggesting that the systems preferentially sampled stable conformations during the simulations.

Collectively, these results demonstrate that all three protein–ligand complexes reached equilibrium and maintained stable structural and energetic properties throughout the production phase of the 300 ns simulations, which is consistent with the minimal changes observed in binding site geometry before and after MD simulations.

### 2.8. Binding Free Energy Analysis

**Figure 9:**
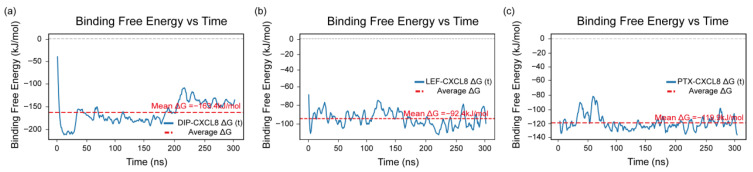
Binding free energy profiles of CXCL8 complexes obtained from MM-PBSA analysis. (**a**) DIP–CXCL8 complex, (**b**) LEF–CXCL8 complex, and (**c**) PTX–CXCL8 complex. The blue lines represent the time-dependent variations in binding free energy (ΔG) over the 300 ns simulation, while the red dashed lines indicate the corresponding average binding free energy values for each complex.

The binding free energy of each complex was calculated using the MM-PBSA method over the entire 300 ns molecular dynamics trajectory to assess the stability and affinity of the protein–ligand interactions. As shown in Figure 9a–c, the mean binding free energies of the DIP–CXCL8, LEF–CXCL8, and PTX–CXCL8 complexes were −163.4, −92.4, and −119.9 kJ/mol, respectively.

The MM-PBSA analysis indicated that all three ligands formed energetically favorable and stable complexes with CXCL8. Among them, DIP exhibited the most negative binding free energy, indicating the strongest binding affinity and the highest complex stability over the 300 ns simulation period. LEF and PTX also maintained consistent interactions with CXCL8, though with relatively weaker binding energies, indicating moderate but stable associations.

Collectively, the results of the MM-PBSA analysis indicate that DIP, LEF, and PTX have the potential to act as CXCL8 modulators and their interactions with CXCL8 may also influence the development and progression of paroxysmal nocturnal hemoglobinuria by modulating CXCL8-related inflammatory and immune pathways.

### 2.9. Cell Viability Assays

**Figure 10:**
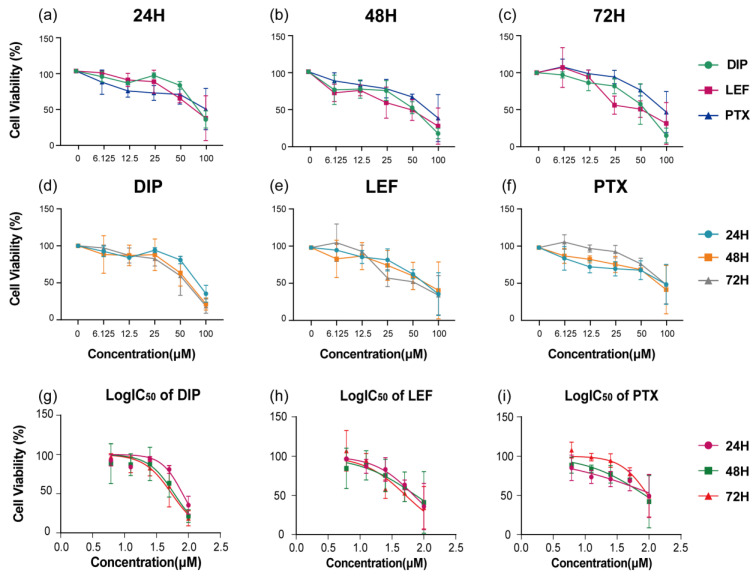
Cell viability of *PIGA*-KO cells treated with DIP, LEF, and PTX at various concentrations and time points. (**a**–**c**) Cell viability after exposure to different concentrations of DIP, LEF, and PTX for 24 h, 48 h, and 72 h, respectively. (**d**–**f**) Comparison of time-dependent cytotoxic effects for each compound. The bottom panels illustrate the corresponding log-transformed IC_50_ curves for (**g**) DIP, (**h**) LEF, and (**i**) PTX, showing a concentration- and time-dependent reduction in cell viability.

The cytotoxic effects of DIP, LEF, and PTX on the *PIGA*-KO cell line were quantitatively evaluated using the CCK-8 assay after 24, 48, and 72 h of treatment. As shown in Figure 10a–c, all three compounds exhibited a concentration- and time-dependent reduction in cell viability. At lower concentrations, minimal cytotoxicity was observed, whereas progressive inhibition of cell proliferation became evident with increasing drug concentrations and prolonged exposure.

A noticeable enhancement in growth inhibition was observed after 72 h compared with the 24 h and 48 h treatments, suggesting a cumulative cytotoxic effect over time. Among the tested agents, LEF demonstrated relatively higher potency at lower concentrations, whereas PTX showed a delayed but pronounced cytotoxic response at higher doses (Figure 10d–f).

Quantitative analysis of IC_50_ values further confirmed the time-dependent sensitivity of *PIGA*-KO cells to these compounds. The IC_50_ values for DIP, LEF, and PTX were 81.08 µM, 69.68 µM, and 125.40 µM at 24 h; 59.89 µM, 74.60 µM, and 87.28 µM at 48 h; and 54.79 µM, 51.98 µM, and 96.71 µM at 72 h, respectively (Figure 10g–i). These results indicate that *PIGA*-KO cells are susceptible to the antiproliferative activity of all three agents, with a gradual increase in inhibitory potency upon prolonged exposure.

## 3. Discussion

**Figure 11:**
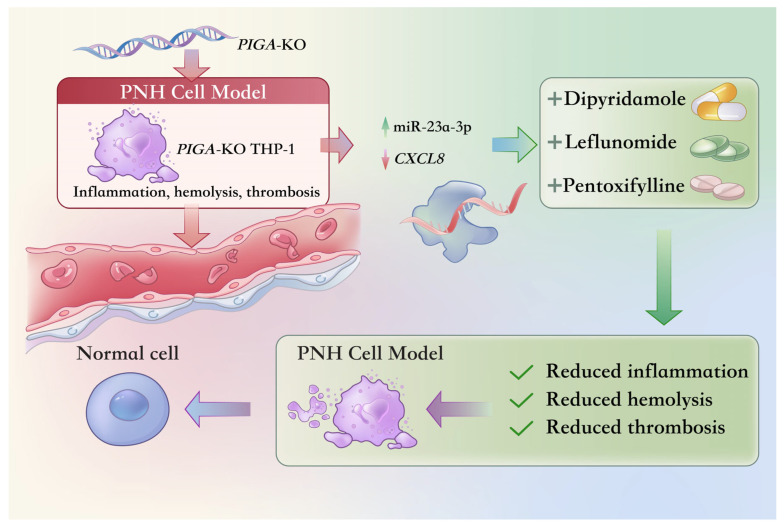
Mechanistic overview of the PNH cell model and therapeutic intervention. This diagram depicts the PNH pathology in a *PIGA*-KO THP-1 cell model, showing the upregulation of miR-23a-3p and the downregulation of *CXCL8*, both of which contribute to inflammation, hemolysis, and thrombosis. Treatment with DIP, PTX, and LEF reverses the expression of these molecules, leading to a reduction in inflammation, hemolysis, and thrombosis. The transition from the PNH cell model to a normal cell state following therapeutic intervention is shown, demonstrating the potential of these drugs to mitigate the clinical manifestations of PNH.

This study systematically investigates the molecular pathogenesis of PNH and potential drug targets, aiming to provide a theoretical basis and data support for better understanding this disorder and developing precision treatment. By constructing a *PIGA*-KO THP-1 cell model and applying various bioinformatics methods, we identified hsa-miR-23a-3p as a core miRNA. It regulates multiple PNH-related signaling pathways through the modulation of *CXCL12*, *CXCL8*, *HES1*, and *TRAF5*, significantly impacting cellular inflammation, chemotaxis, and immune regulation. Furthermore, we extended our study by predicting potential therapeutic drugs that might target the dysregulated pathways identified in the PNH model. Through a comprehensive drug repurposing approach, we selected three promising candidates—DIP, PTX, and LEF. Molecular docking simulations were conducted to assess the binding affinity of these drugs with critical target proteins, revealing favorable interactions and supporting their potential efficacy in mitigating PNH-related symptoms (Figure 11).

Previous studies [23] have reported that in myelodysplastic syndrome (MDS) patients, the expression of *CXCL12* and miR-23a in stromal cells is negatively correlated, and for the first time, the miR-23a/*CXCL12* axis was shown to regulate the hematopoietic microenvironment. Additionally, Liu et al. [24] found that miR-23a-3p regulates *CXCL12* to affect angiogenesis. Research by Moon et al. [25] indicated that although miR-23a-3p does not directly target *CXCL8*, both are part of the same regulatory network, with potential targeting effects that influence inflammation, cytokine cascades, and other cardiovascular disease-related mechanisms. Shang et al. [26] further demonstrated that miR-23a-3p directly binds and inhibits the expression of *TRAF5*, regulating cell survival and proliferation. Collectively, miR-23a-3p plays a significant role in the onset and progression of PNH and related hematological diseases through a multi-target, multi-pathway regulatory mechanism. These regulatory effects correlate with clinical phenomena such as the high incidence of hemolysis, thrombosis, and infections in PNH patients.

Neutrophils, the most abundant white blood cells in blood, are key members of the innate immune system. They primarily develop in the bone marrow and combat pathogens through phagocytosis, degranulation, and the formation of extracellular traps. In addition, neutrophils play multiple roles in tissue repair and fibrosis, protecting the body while potentially contributing to inflammation and tissue damage [27,28]. Clinically, the detection of GPI-AP-deficient neutrophil clones through flow cytometry allows for the sensitive and accurate diagnosis of PNH, including the detection of very small clones, which aids in early disease detection and monitoring. Compared to red blood cells, neutrophils are less affected by hemolysis and other interferences in PNH detection, providing a distinct diagnostic advantage [29]. Neutrophil extracellular traps (NETs), which are web-like structures composed of DNA and antimicrobial proteins released by neutrophils in response to specific stimuli, capture and eliminate pathogens [30]. Numerous studies have shown that while NETs serve to protect the body, they also promote thrombosis and participate in inflammation and tissue damage in various disease states [31,32,33,34]. In this study, single-cell transcriptome analysis not only further confirmed the central role of neutrophils in the immune microenvironment of PNH patients but also revealed their unique role in regulating local inflammation, thrombosis, and complement activation. hdWGCNA analysis identified several co-expression modules and core genes closely associated with neutrophil function, providing strong evidence for further elucidating their molecular action network in PNH pathogenesis.

Building on these findings, we propose an integrative mechanistic model linking neutrophil activation, NET formation, miRNA regulation, and thrombosis in PNH. Single-cell transcriptomic analysis revealed a neutrophil population enriched for inflammatory and NET-associated gene expression, suggesting a heightened propensity for NET release. NETs are known to expose extracellular DNA, histones, and granular proteins that not only amplify local inflammation but also provide a pro-thrombotic scaffold that promotes platelet adhesion, coagulation cascade activation, and endothelial injury.

In our study, single-cell transcriptomic analysis confirmed the central role of neutrophils in the immune microenvironment of PNH patients. We observed that neutrophils in PNH are enriched for genes associated with inflammation, thrombosis, and NET formation. These findings suggest that neutrophils in PNH are primed for excessive activation, contributing to local inflammation, endothelial injury, and thrombotic risk. We propose an integrative mechanistic model linking neutrophil activation, NET formation, miRNA regulation, and thrombosis in PNH. This model suggests that miR-23a-3p plays a crucial role in modulating neutrophil function. Dysregulation of miR-23a-3p expression can potentially enhance neutrophil activation and NET formation, thereby increasing thrombotic risk in PNH.

Together, these results support an NET-centered pathogenic framework for PNH, where dysregulated neutrophil activation, miRNA-mediated regulatory imbalance, and thrombo-inflammatory processes converge to drive disease progression. Although further experimental validation is required, this integrative model offers a coherent explanation that bridges single-cell transcriptomic findings with molecular regulation and clinical phenotypes in PNH.

In terms of drug repositioning, in this study, based on multi-omics data, combined with drug repositioning methods, bioinformatics predictions, clinical guidelines, molecular docking, and molecular dynamics simulations, we identified potential therapeutic drugs for PNH. Through intersection analysis of the predictions from multiple drug platforms, eight candidate drugs were finally selected: Prednisone, Tretinoin, Dipyridamole, Danazol, Pentoxifylline, Leflunomide, Colchicine, and Paclitaxel. This study further evaluated the feasibility of these drugs by considering their existing clinical applications, pharmacological mechanisms, and safety profiles.

Prednisone, a corticosteroid, is widely used in the clinical treatment of PNH and is recommended as a first-line therapy in mainstream guidelines [35,36]. It mainly alleviates acute episodes by inhibiting inflammatory responses and immune-mediated hemolysis [37,38]. Tretinoin has been shown to have some relieving effects in PNH [39]. Danazol, a synthetic androgen, promotes hematopoiesis but imposes a significant burden on the liver and carries a higher risk of complications with long-term use [40,41,42]. Colchicine and Paclitaxel are primarily used for malignant tumors and autoimmune diseases; the former has noticeable toxic side effects as a traditional chemotherapeutic agent [43], and the latter, while capable of interfering with microtubule polymerization, has higher toxicity, and there is insufficient evidence supporting its clinical feasibility in PNH [44]. Therefore, neither Colchicine nor Paclitaxel is considered a suitable candidate for PNH treatment.

Based on an in-depth examination of drug mechanisms and clinical needs, LEF, DIP, and PTX are considered to have greater translational potential. LEF, a classic immunomodulator, is widely used in autoimmune diseases such as rheumatoid arthritis [45]. It works by inhibiting dihydroorotate dehydrogenase, blocking pyrimidine synthesis, and significantly suppressing the activation and proliferation of T and B lymphocytes [46,47]. Moreover, LEF downregulates the expression of various inflammatory factors, including CXCL8 (IL-8), which can effectively reduce complement-mediated hemolysis and associated inflammatory responses [48,49]. The molecular docking and molecular dynamics simulation results of this study further confirm that LEF has notable binding ability with core miRNAs and genes, theoretically providing an effective intervention for immune and inflammatory abnormalities in PNH, offering a molecular basis for clinical treatment.

DIP is a classic anti-platelet aggregation drug that can dilate blood vessels [50], increase microcirculatory perfusion, and improve tissue hypoxia, with relatively low bleeding risk and mild side effects [51,52]. In PNH patients, due to vascular damage and high thrombosis risk caused by intravascular hemolysis and complement activation, the rational application of anti-platelet aggregation drugs is crucial for prevention and treatment. Molecular docking and dynamics simulation results in this study also indicate that DIP can effectively bind and regulate multiple PNH-related target molecules, providing a molecular basis for inhibiting thrombosis and improving microcirculation.

PTX is a drug that improves microcirculation [53], primarily used in peripheral vascular and chronic ischemic diseases. PTX inhibits phosphodiesterase, thereby reducing the synthesis of inflammatory factors [54,55,56,57]. It can also lower blood viscosity, enhance red blood cell deformability, inhibit platelet aggregation, and modulate the release of inflammatory factors [58,59]. In the molecular docking and dynamics simulations carried out in this study, PTX showed good binding ability with key inflammatory signal molecules such as CXCL8, theoretically helping to improve blood rheology and reduce vascular damage following hemolysis in PNH patients.

In addition, the results from the CCK-8 assay demonstrated that LEF, DIP, and PTX exhibited significant cytotoxic effects on *PIGA*-KO cells. These findings suggest that LEF, DIP, and PTX may serve as potential therapeutic candidates for PNH, particularly through their roles in complement inhibition, the modulation of inflammatory responses, and the prevention of thrombosis.

Despite the valuable insights gained, this study has several limitations that should be acknowledged. Firstly, while the *PIGA*-KO THP-1 cell model was effectively constructed using CRISPR/RNP technology to simulate the molecular pathology of PNH, it is important to recognize the inherent differences between the cell model and the complex in vivo physiological environment. This discrepancy limits the full translatability of our findings to actual clinical scenarios.

Secondly, although bioinformatics methods, molecular docking, and molecular dynamics simulations were employed to identify key regulatory targets and signaling pathways, certain predicted targets and their specific biological functions in PNH pathogenesis are still to be experimentally validated. While these computational approaches provide valuable hypotheses, direct experimental validation is crucial in confirming their role in disease mechanisms.

Furthermore, although multiple platforms and algorithms were used to minimize false-positive rates in drug prediction, the candidate drugs identified may still face significant challenges in real-world clinical applications. Issues such as safety, optimal dosing, drug interactions, and potential side effects must be addressed before any clinical implementation takes place.

Additionally, while this study revealed encouraging interaction profiles for the compounds, the absence of a reference ligand or positive control limits the ability to directly compare the binding affinity of the compounds. This limitation reflects the current clinical landscape, as no approved or well-established CXCL8 targeting small-molecule drugs is available. It is also important to note that CXCL8 primarily exerts its biological function through protein–protein interactions with the receptors CXCR1 and CXCR2, which differ fundamentally from protein–small molecule interactions. Therefore, the compounds identified should be regarded as promising CXCL8-interacting candidates rather than definitive inhibitors. Although molecular dynamics simulations suggest that DIP, LEF, and PTX may modulate CXCL8 activity, further investigation into the precise binding mechanisms and their therapeutic relevance in the context of PNH is needed. Experimental validation, including biochemical, cellular, and in vivo studies, is essential to fully understand the biological effects and clinical potential of these compounds.

Lastly, while single-cell transcriptomics and hdWGCNA analyses provided valuable insights into neutrophil-related gene expression changes and suggested the potential involvement of neutrophils in inflammation, thrombosis, and complement activation in PNH, direct functional validation was not performed. Specifically, experimental evidence of neutrophil extracellular trap (NET) formation, pro-thrombotic activity, or complement-mediated neutrophil dysfunction in patient samples was not assessed. Therefore, the proposed roles of neutrophils and NETs in PNH should be considered hypothesis-generating, and additional studies integrating functional assays, such as NET detection, flow cytometric analysis of neutrophil activation, and thrombosis models, are needed to confirm and expand upon these findings.

## 4. Materials and Methods

### 4.1. Materials

The THP-1 cell line was purchased from Tsingke Biotechnology Co., Ltd. (Beijing, China). RPMI 1640 medium, fetal bovine serum (FBS), phosphate-buffered saline (PBS), and penicillin–streptomycin solution were purchased from Gibco (Waltham, MA, USA). RNA extraction kits, the FastPure^®^ Cell/Tissue Total RNA Isolation Kit V2, the HiScript^®^ III All-in-one RT SuperMix Perfect for qPCR, and the Taq Pro Universal SYBR qPCR Master Mix were purchased from Vazyme Co., Ltd. (Nanjing, China). The primers required for RT-qPCR experiments were synthesized by Biomed Technology Co., Ltd. (Beijing, China). CCK8 was obtained from Dojindo Laboratories (Tokyo, Japan).

### 4.2. Construction of Gene Knockout Cell Line Using CRISPR/RNP Method

THP-1 cells, a human monocytic cell line, are commonly used to model immune processes, including inflammation and cytokine production, which are central in the pathophysiology of PNH. While PNH is primarily characterized by complement-mediated hemolysis and thrombosis, THP-1 cells provide crucial insights into the immune dysregulation associated with the disease, making them an invaluable tool for studying its inflammatory components.

In this study, *PIGA* gene knockout was performed in THP-1 cells using a Cas9-based CRISPR/RNP system [60]. Target sequences were selected based on functional regions and low off-target principles, and two sgRNAs (AGAUACCAUGCAUAUAUUAU and ACACUCUCUCGGGUUAGCCC) were designed. The sgRNAs and Cas9 protein were mixed at a 5:1 to 10:1 ratio to form the RNP complex, which was then transfected into cells using the Neon NxT electroporation system. After electroporation, single-cell sorting and amplification were performed, and single clones with high knockout efficiency and good condition were selected and preserved. Gene knockout efficiency was assessed via PCR. Cells were cultured under standard conditions (RPMI-1640, 10% FBS, 1% P/S, 37 °C, 5% CO_2_), and all experiments were conducted according to the manufacturer’s instructions.

Gene knockout experiments were outsourced to Tsingke Biotechnology Co., Ltd (Beijing, China).

### 4.3. Validation of Cell Line Using Molecular Biology Techniques and Whole-Transcriptome Sequencing

Total RNA was extracted from *PIGA*-KO THP-1 cells and wild-type (WT) THP-1 cells using the FastPure^®^ Cell/Tissue Total RNA Isolation Kit V2. The RNA purity and concentration were assessed using a NanoDrop spectrophotometer (Thermo Scientific, Waltham, MA, USA). Reverse transcription was performed using the HiScript^®^ III All-in-one RT SuperMix Perfect for qPCR, and cDNA amplification was carried out using the Taq Pro Universal SYBR qPCR Master Mix. The primers for the *PIGA* were as follows: upstream primer sequence, CCTGTAGAGGAGGAGCTGGGAATGG; downstream primer sequence, CACCGAGCTGACATCAGCAAA. The primers for the β-actin were as follows: upstream primer sequence, CTACAATGAGCTGCGTGTGGC; downstream primer sequence, CAGGTCCAGACGCAGGATGGC. The relative expression of the target gene was calculated using the 2^−ΔΔCt^ method.

Total RNA was extracted using Trizol reagent, and whole-transcriptome sequencing was performed by Tsingke Biotechnology Co., Ltd. (Beijing, China), using the Illumina platform.

### 4.4. Upstream Analysis and Differential Expression Analysis

An analysis platform was constructed in a Linux environment, where tools such as FastQC, MultiQC, Samtools, HISAT2, RSeQC, and Bowtie2 were installed. miRNA and mRNA data were preprocessed, aligned, and quantified using miRDeep2 [61] and HISAT2, respectively. The miRbase (https://www.mirbase.org/, accessed on 1 August 2025) and Ensembl (https://www.ensembl.org/index.html, accessed on 1 August 2025) were used to obtain the miRNA and mRNA expression profiles for each sample, which were used for subsequent analysis. The ensembl_gene_id format from the upstream quantification results was converted to the hgnc_symbol format. Differential expression analysis of the mRNA and miRNA quantification results was performed using the DESeq2 package (version 1.48.2) in R 4.4.1. Differential expression mRNAs (DEmRNAs) and differential expression miRNAs (DEmiRNAs) were identified based on *p* < 0.05 and |Log_2_FC| > 1. Finally, the results were visualized using the ggplot2 package (version 4.0.1) in R 4.4.1.

### 4.5. Functional Enrichment Analysis and Gene Set Enrichment Analysis

The differentially expressed gene names filtered based on threshold were converted to the entrezgene_id format. Using the clusterProfiler package (version 4.16) in R 4.4.1, Gene Ontology (GO) and Kyoto Encyclopedia of Genes and Genomes (KEGG) functional annotation was performed, with *p* < 0.05 as the threshold.

### 4.6. Construction of miRNA-mRNA Regulatory Axis and Identification of Key Genes

The multiMiR package (version 3.22) [62] in R 4.4.1 was used to predict and select target genes validated via dual-luciferase reporter assays. The regulatory direction was determined based on the sign of Log_2_FC, and the intersection with DEmRNAs was further analyzed to construct the miRNA–mRNA regulatory network. ENCORI (https://rnasysu.com/encori/, accessed on 1 August 2025) was used to predict the enriched pathways of DEmiRNAs. miRNAs enriched in multiple PNH-related pathways were considered core miRNAs, and their regulated target genes were identified as core genes in PNH.

### 4.7. Single-Cell RNA-Seq Analysis

A search string was conducted in PubMed (https://pubmed.ncbi.nlm.nih.gov/, accessed on 5 August 2025) using the query (“PNH” [Title]) AND (“multiomics” [Title]), and single-cell RNA sequencing (scRNA-seq) data for PNH from relevant publications were downloaded as the disease group. The data were processed with Cell Ranger 9.0.1 for upstream quantification analysis. Normal human scRNA-seq data were retrieved from DISCO [63] (https://disco.bii.a-star.edu.sg/, accessed on 5 August 2025) as the control group. The filtering criteria for cells and genes were as follows: for the disease group, cells with fewer than 30,000 expressed genes, fewer than 5000 genes, and less than 8% mitochondrial gene content were retained; for the control group, cells with fewer than 10,000 expressed genes, fewer than 2000 genes, and less than 15% mitochondrial gene content were retained. After integrating the scRNA-seq data from the disease and control groups, data were normalized using the NormalizeData function. Batch effect correction was performed using the Harmony function for disease and normal samples, followed by principal component analysis (PCA) to compute the principal components of the data. K-nearest neighbor (KNN) clustering analysis was then applied to the top 30 principal components selected from the data. Finally, cell types were annotated using CellMarker 2.0 (http://bio-bigdata.hrbmu.edu.cn/CellMarker/, accessed on 7 August 2025) and ACT (http://xteam.xbio.top/ACT/index.jsp, accessed on 7 August 2025). Cell communication was analyzed using the CellChat package (version 2.2.0) in R 4.4.1, with the computeCommunProb function and the population.size parameter to normalize cell abundance across clusters. Enrichment analysis focused on PNH-related cell types, including monocytes, macrophages, megakaryocytes, and neutrophils. High-dimensional Weighted Gene Co-expression Network Analysis (hdWGCNA) was used to identify the modules most highly associated with the neutrophil cluster, highlighting key modules for further investigation. Additionally, the expression of *PIGA* in neutrophils was validated to confirm its relevance in the context of PNH.

### 4.8. Prediction of Potential Therapeutic Drugs for PNH

Using the Epigenomic Precision Medicine Prediction Platform (EpiMed) [64], along with the CMap [65] (https://clue.io/query, accessed on 10 August 2025), DEmRNAs were used as input for drug prediction. Core genes in the miRNA-mRNA regulatory network associated with PNH were input into the DGIdb [66] (https://dgidb.org/, accessed on 10 August 2025) for drug prediction. The intersection of key modules obtained from hdWGCNA analysis and DEmRNAs in the neutrophil cluster was also used as input for drug prediction in the CMap. The drug prediction results from these four sources was analyzed to identify potential therapeutic drugs for PNH.

### 4.9. Molecular Docking Analysis

The 3D structure files of the proteins encoded by core genes were downloaded from the PDB (https://www.rcsb.org/, accessed on 12 August 2025) and the protein structures (PDB ID:6N2U) were examined using PyMOL 3.1 for subsequent docking. SDF format files of potential therapeutic drugs were downloaded from the PubChem (https://pubchem.ncbi.nlm.nih.gov/, accessed on 10 August 2025), and converted into PDB format files using OpenBabel 3.1.1. AutoDock Tools 1.5.7 was used to add hydrogen atoms to the core gene-encoded proteins, as well as to hydrogenate and define rotatable bonds for the small molecule drugs, saving the files in PDBQT format. The docking parameters were set using the Grid module, with semi-flexible docking specified, an exhaustiveness of 8, and the Lamarckian genetic algorithm as the docking algorithm. MD was performed using AutoDock Vina 1.2.0, yielding binding free energies and docking result files. Finally, visualization was performed using PyMOL 3.1 and BIOVIA Discovery Studio.

### 4.10. Molecular Dynamics Simulation Analysis

MDS were performed on the protein-ligand complexes obtained from MD. The complexes were preprocessed and split using PyMOL 3.1. Subsequently, the topology files of the drug molecules were generated using sobtop 1.0 based on the AMBER force field. The topology files for the protein were created using GROMACS (version 2022.5) with the amber99sb-ildn.ff force field. The system, including ions, solvent, receptor, and ligand, was preprocessed in three main steps. First, energy minimization was performed using the steepest descent algorithm to stabilize the system. Then, during heating to 300 K, position restraints were applied to both the receptor and the ligand in each system. Finally, MDS were performed under constant pressure (1 atm) and temperature (300 K), using the Number of particles, Pressure, and Temperature ensemble. After a 300 ns MDS, various dynamic parameters were calculated using the built-in scripts of GROMACS, including root mean square deviation (RMSD), root mean square fluctuation (RMSF), radius of gyration (Rg), solvent accessible surface area (SASA), hydrogen bonds, interatomic distances, and Gibbs free energy.

### 4.11. Binding Free Energy Calculations

The binding free energies between the protein and ligand were estimated using the molecular mechanics Poisson–Boltzmann surface area (MM-PBSA) method which combines molecular mechanics (MM) energies with solvation free energy contributions. The calculations were performed with the g_mmpbsa tool integrated in the GROMACS (version 2022.5). A 300 ns MDS was conducted for each protein–ligand complex, and MM-PBSA analyses were performed throughout the entire 300 ns trajectory to estimate the binding free energy [67].

The MM energy components, including van der Waals and electrostatic interactions, were directly obtained from the GROMACS force field. The polar solvation free energy was calculated by numerically solving the Poisson–Boltzmann equation using the Adaptive Poisson–Boltzmann Solver (APBS) program. The solvent dielectric constant and solute dielectric constant were set to 80 and 2, respectively, under the nonlinear Poisson–Boltzmann (npbe) model at 298 K.

### 4.12. Cell Viability Assay

Cell viability following drug treatment was quantitatively assessed using the Cell Counting Kit-8 (CCK-8) assay in the *PIGA*-KO THP-1 cell line. A total of 5 × 10^5^ viable cells from two cell lines were seeded in 96-well plates, with three replicate wells per group, to ensure uniformity in experimental conditions. The cells were incubated at 37 °C with 5% CO_2_ for 2 h in a constant-temperature incubator, followed by treatment with various concentrations of the drugs (6.25, 12.5, 25, 50, and 100 μM) for 24, 48, and 72 h. After treatment, 20 μL of CCK-8 solution was added to each well and incubated for 2 h at 37 °C with 5% CO_2_. The optical density (OD) at 450 nm was measured using a microplate reader to determine cell viability. Cell survival rates were calculated as follow:

$$\mathrm{cell}\;\mathrm{survival}\;\mathrm{rate}(\%)=\frac{{\mathrm{OD}}_{\mathrm{control}}-{\mathrm{OD}}_{\mathrm{test}}}{{\mathrm{OD}}_{\mathrm{control}}-{\mathrm{OD}}_{\mathrm{blank}}}\times 100\%$$

Based on the cell survival rate, the half-maximal inhibitory concentration (IC_50_) was calculated to evaluate the cytotoxicity of the compound.

### 4.13. Statistical Analysis

All data analyses and visualizations were performed using R 4.4.1 and GraphPad Prism 8.0. An unpaired *t*-test was used for comparisons between two groups. Experiments were conducted with at least three biological replicates. In bioinformatics analysis, a statistical significance threshold of *p* < 0.05 and |Log_2_FC| > 1 were considered statistically significant.

## 5. Conclusions

This study proposes a potential mechanism for the molecular pathogenesis of PNH through the establishment of a *PIGA*-KO THP-1 cell model and integrative bioinformatics analyses. It identifies the miR-23a-3p/*CXCL8* axis as a pivotal regulatory pathway that orchestrates multiple signaling pathways involved in inflammation, chemotaxis, and immune regulation. These findings provide mechanistic insights into the hallmark clinical manifestations of PNH, including hemolysis, thrombosis, and increased susceptibility to infections. Single-cell transcriptomics further emphasizes the central role of neutrophils in the PNH immune microenvironment, linking their pro-inflammatory and pro-thrombotic activities to disease progression.

Drug repurposing (DR) analyses, supported by molecular docking (MD) and molecular dynamics simulations (MDSs), identified Leflunomide, Dipyridamole, and Pentoxifylline as promising therapeutic candidates with the potential to modulate complement activation, inflammation, and thrombosis. While these findings provide a strong theoretical foundation for precision treatment strategies, several limitations must be acknowledged. Firstly, this study did not incorporate additional gene knockout models for further experimental validation. Secondly, further experiments are needed to verify the effects of these drugs on cellular activity and to confirm the regulatory relationship between miR-23a-3p and *CXCL8*.

Moreover, this research primarily serves as a reference for clinical applications, with the proposed drugs still in the prediction phase. Extensive experimental validation is required to confirm their efficacy and clinical relevance. Future studies integrating clinical trials and mechanistic experiments will be essential to substantiate these therapeutic prospects and advance personalized management strategies for PNH.

## Figures and Tables

**Table 1:** Node Information and Regulatory Directions in the miRNA-mRNA Regulatory Network.

miRNA	Target Gene	Regulatory Direction of miRNA
hsa-miR-129-1-3p	*BDKRB2*	up
hsa-miR-143-3p	*HK2 HRAS*	up
hsa-miR-181d-5p	*HRAS*	up
hsa-miR-193a-3p	*CCND1*	up
hsa-miR-193a-5p	*IGF2BP1*	up
hsa-miR-22-5p	*GSDMD*	up
hsa-miR-23a-3p	*CXCL12 CXCL8 HES1 TRAF5*	up
hsa-miR-34b-3p	*CCND1*	up
hsa-miR-365a-3p	*CCND1*	up
hsa-miR-95-3p	*CCND1*	up
hsa-miR-144-5p	*MET*	down
hsa-miR-146b-5p	*A2M EGFR ERBB4*	down
hsa-miR-151a-3p	*TWIST1*	down
hsa-miR-182-5p	*ADCY6 ARRDC3 CCND2 PDK4 THBS1*	down
hsa-miR-183-5p	*EGR1*	down
hsa-miR-216b-5p	*KRAS*	down
hsa-miR-217-5p	*KRAS*	down
hsa-miR-224-5p	*DNM1L FOSB RASSF8 TRIB1*	down
hsa-miR-30a-3p	*CCN1 THBS1*	down
hsa-miR-30c-1-3p	*NR1I2*	down
hsa-miR-30c-5p	*CAMK2D CCN2 CCND2*	down
hsa-miR-34a-5p	*ANK3 AXL KIT KLF12 LEF1 MET NR4A2*	down
hsa-miR-484	*KLF12*	down
hsa-miR-506-3p	*BCAT1*	down
hsa-miR-509-3p	*MAP3K8*	down
hsa-miR-96-5p	*ADCY6 KRAS*	down

**Table 2:** Intersection of drugs from drug sets A, B, C, and D.

Drug Name	CID	Pharmacological Class	Target Gene
Prednisone	5865	Glucocorticoid	*CXCL12*
Tretinoin	444795	Retinoid	*HES1*, *CXCL8*
Dipyridamole	3108	Antiplatelet	*CXCL8*
Danazol	28417	Androgen	*CXCL8*
Pentoxifylline	4740	Vasodilator	*CXCL8*
Leflunomide	3899	Immunosuppressant	*CXCL8*
Colchicine	6167	Anti-inflammatory	*CXCL8*
Paclitaxel	36314	Chemotherapeutic	*CXCL8*

## Data Availability

The original data presented in this study are openly available in a publicly accessible repository. Specifically, the processed data are available on GitHub at https://github.com/lulab-pz/PNH_bioinformatics_analysis. The raw sequencing data are available in the NCBI BioProject under accession number PRJNA1305106 (accessed on 5 August 2025).

## Supplementary Materials

The following supporting information can be downloaded at: https://www.mdpi.com/article/10.3390/ph19010143/s1. Supplementary Figure S1: RT-qPCR validation of *PIGA* gene expression in *PIGA*-KO THP-1 cells and wild-type (WT) cells, based on three independent biological replicates. Supplementary Figure S2: Volcano plot of differentially expressed genes in neutrophils from the single-cell RNA-seq dataset. Supplementary Figure S3: Differential expression of the *PIGA* gene in neutrophils between disease and control groups in the single-cell RNA-seq dataset. Supplementary Table S1: Relative expression levels of the *PIGA* gene in *PIGA*-KO THP-1 cells and WT cells as determined by RT-qPCR. Supplementary Table S2: Results of differential expression analysis for mRNA and miRNA. Supplementary Table S3: GO and KEGG enrichment analysis results of mRNA sequencing data. Supplementary Table S4: Target gene information of differentially expressed miRNAs (DEmiRNAs). Supplementary Table S5: Pathway enrichment prediction results for miRNAs. Supplementary Table S6: Pearson correlation analysis between *PIGA*-KO THP-1 cells and WT cells. Supplementary Table S7: Differential expression analysis results of neutrophils in the single-cell RNA-seq dataset. Supplementary Table S8: Intersection of DEmRNAs and neutrophil-associated gene sets identified by hdWGCNA. Supplementary Table S9: Intersection results among Drug Sets A, B, C, and D.
