# Supplementary figures and images for: Integrative miRNA–mRNA Network and Molecular Dynamics-Based Identification of Therapeutic Candidates for Paroxysmal Nocturnal Hemoglobinuria
Source: Pharmaceuticals (Basel). 2026 Jan 14;19(1):143. doi: 10.3390/ph19010143 (PMC12845513; doi:10.3390/ph19010143)

Relative expression of *PIGA*

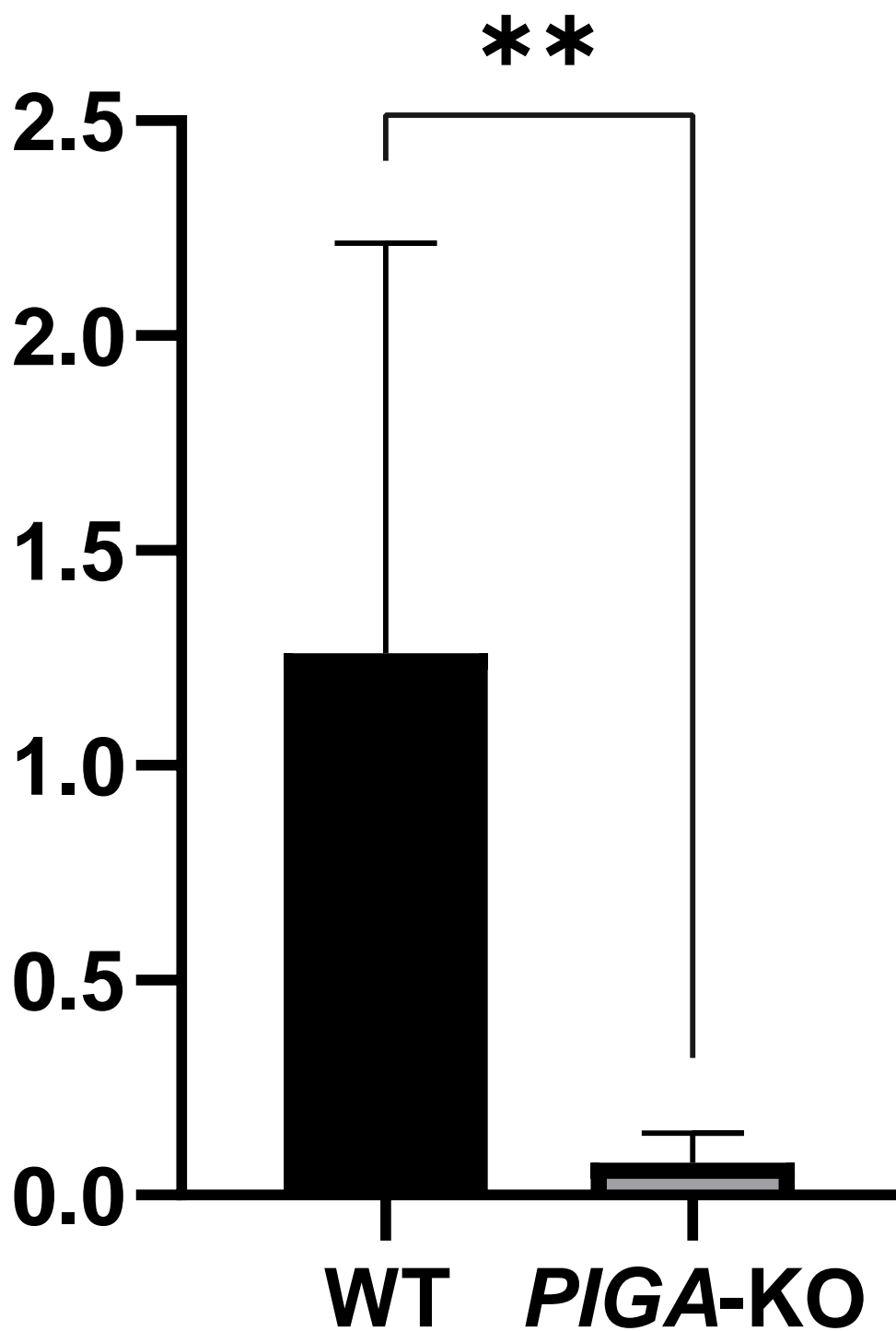

**Relative expression of *PIGA***

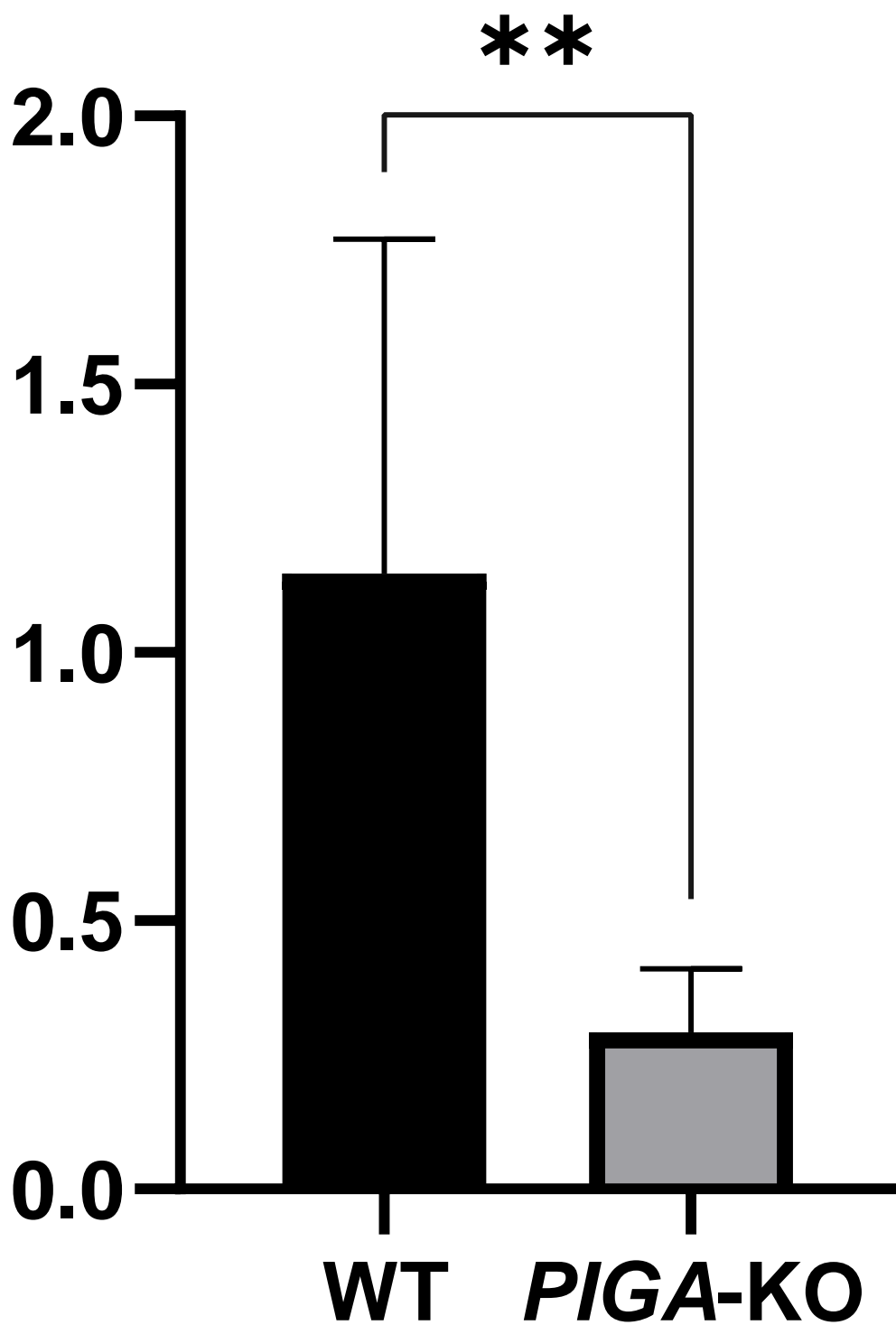

Relative expression of *PIGA*

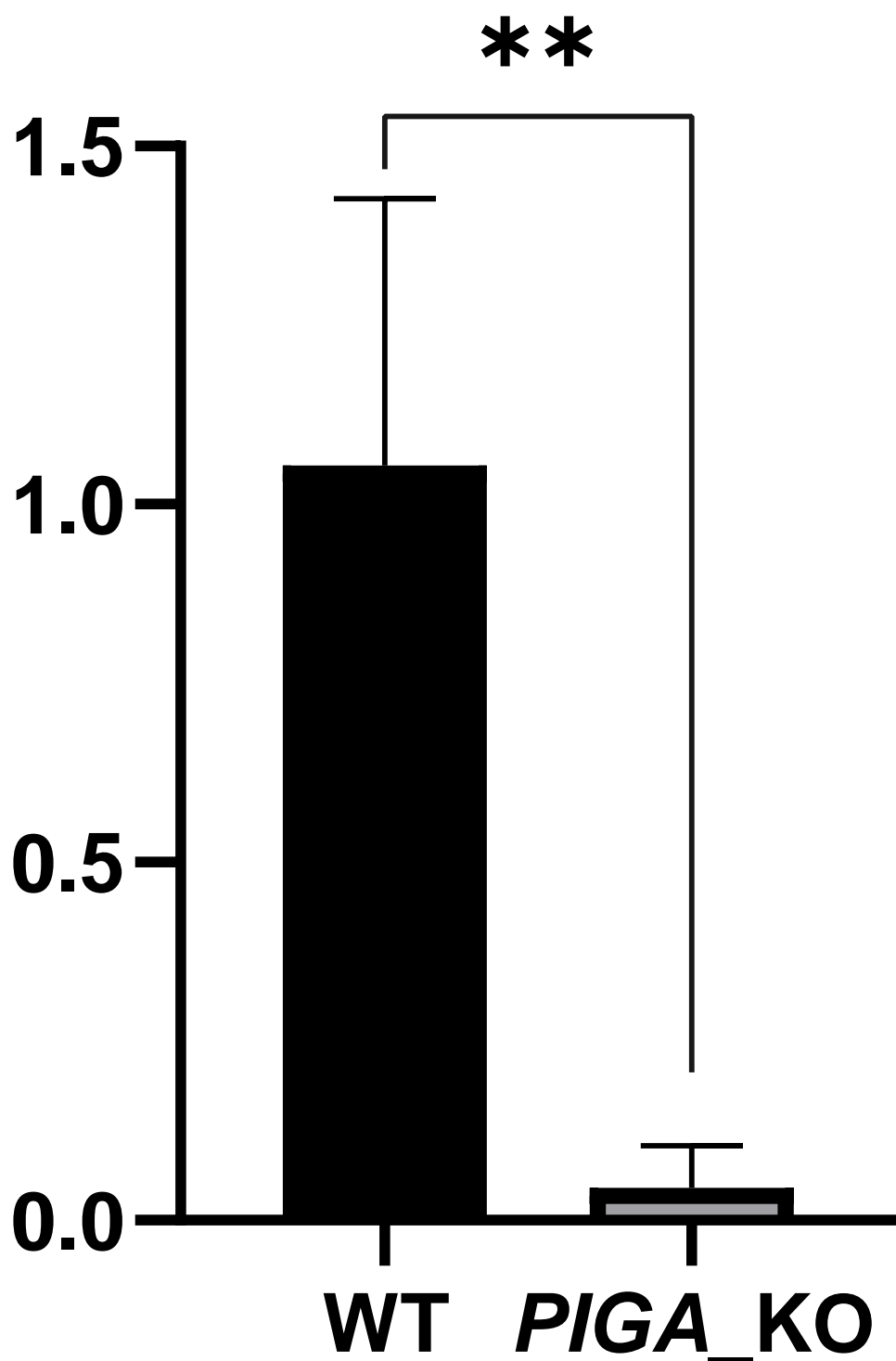

Supplement: Supplementary file 1 [file pharmaceuticals-19-00143-s001.zip › Supplementary_Figure_S1.pdf]

# mRNA

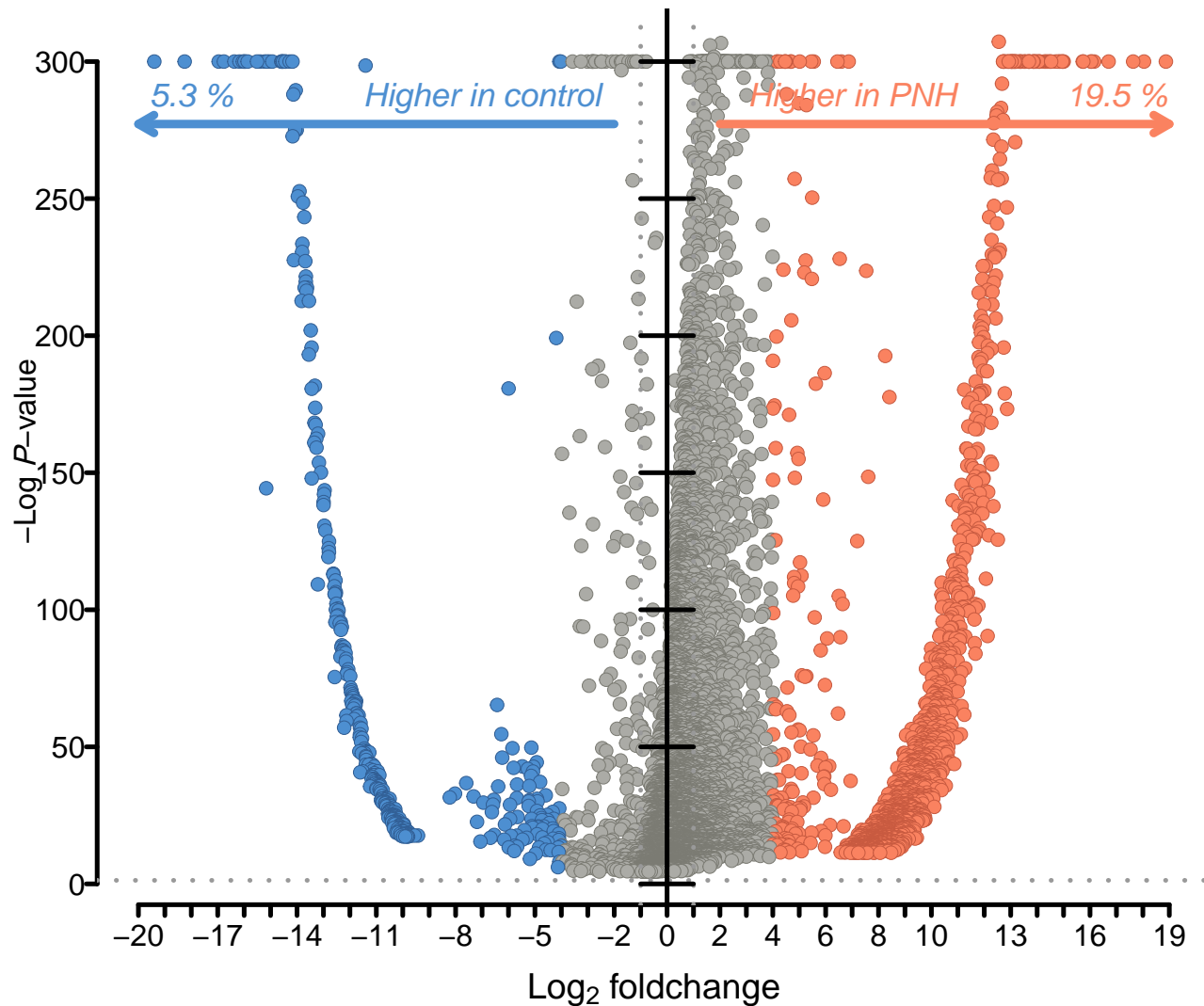

Supplement: Supplementary file 1 [file pharmaceuticals-19-00143-s001.zip › Supplementary_Figure_S2.pdf]

PIGA expression in neutrophils

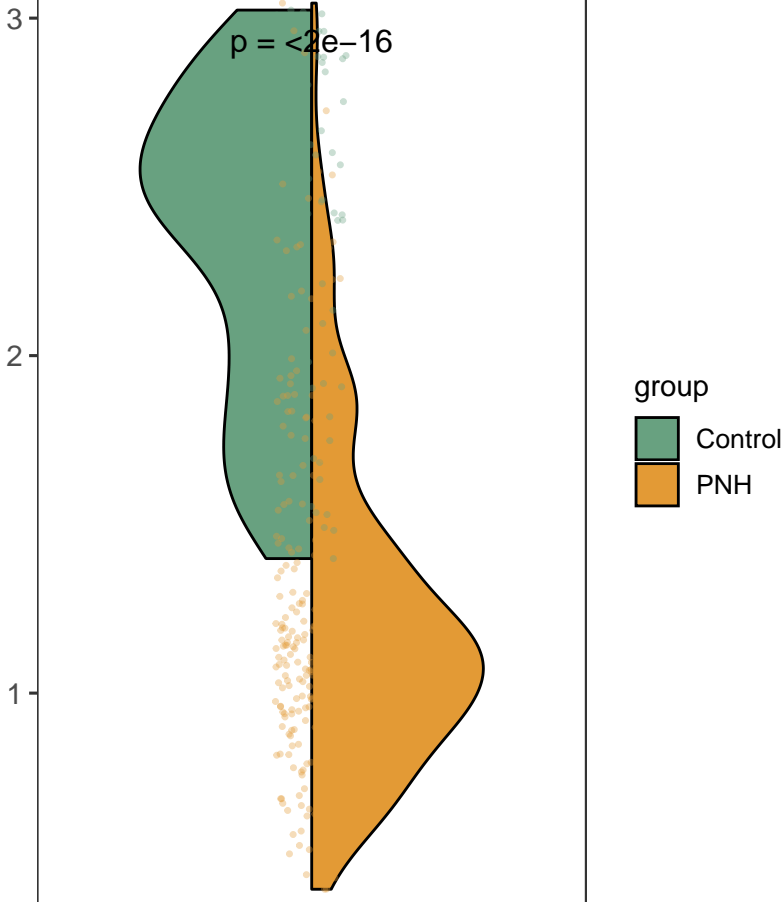

Supplement: Supplementary file 1 [file pharmaceuticals-19-00143-s001.zip › Supplementary_Figure_S3.pdf]
